# Comparative study of functional outcomes between OTA/AO type C, Gustilo type I/II open fractures and closed fractures of the distal humerus treated by open reduction and internal fixation

**DOI:** 10.1186/s12891-021-04817-1

**Published:** 2021-11-10

**Authors:** Chen Chen, Dan Xiao, Ting Li, Maoqi Gong, Yejun Zha, Kehan Hua, Weitong Sun, Shangwei Ji, Xieyuan Jiang

**Affiliations:** Department of Orthopedic Trauma, Beijing Jishuitan Hospital, No.31 Xinjiekou East Street, Xicheng District, Beijing, 100035 China

**Keywords:** Open fracture, Gustilo type I/II, Distal humeral fracture, Open reduction and internal fixation

## Abstract

**Background:**

To evaluate the difference of functional outcomes between OTA/AO type C, Gustilo type I/II open fractures and closed fractures of the distal humerus after open reduction and internal fixation.

**Methods:**

We retrospectively analyzed the clinical data of patients with OTA/AO-C distal humerus fractures who were treated in our department from January 2014 to December 2016. The patients were divided into an open fracture group and a closed fracture group. Their baseline characteristics and functional outcomes were analyzed and compared.

**Results:**

A total of 64 patients treated by operative fixation were identified (25 open and 39 closed injuries), and the average follow-up time was 35.1 ± 13.6 months. There were no significant differences in the range of motion (ROM) of the elbow, Mayo Elbow Performance Score (MEPS), Disabilities of the Arm, Shoulder and Hand (DASH) score, complications, hospitalization time, operation time, intraoperative blood loss, or medical costs between the two groups (*P* > 0.05).

**Conclusion:**

OTA/AO type C, Gustilo I/II distal humeral open fractures can yield satisfactory clinical results similar to those of closed distal humeral fractures after open reduction and internal fixation.

**Level of evidence:**

Therapeutic Level III; Retrospective Cohort Comparison; Treatment Study.

## Background

The incidence of distal humerus fractures is approximately 5.7 cases per 100,000 per year [1, 2]. Most adult distal humerus fractures (96%) are OTA/AO-C fractures, which are often resulted from high-energy trauma [3]. This type of fracture was difficult to treat due to comminuted articular surface and poor soft tissue conditions [4]. With the advancement of surgical technique in recent years, many studies have shown that open reduction and internal fixation (ORIF) can yield good outcomes in distal humerus fractures [5–9].

However, open distal humerus fractures are still a great challenge for orthopedic surgeons to manage owing to its fracture fragments penetrating the soft tissue of the elbow, which leads to severe soft tissue injury and often result in deep infection, fracture nonunion, and elbow stiffness [10]. The Gustilo-Anderson classification system is universally used for open fractures [11]. Gustilo type I/II fractures are low-energy injuries, with minimal soft tissue injuries, and type III fractures are high-energy injuries that are often accompanied by extensive soft tissue injuries [12, 13].

Most of the existing studies focused on open distal humerus fracture treatments alone, without further evaluating the subtypes of Gustilo classes [5, 14]. The treatment methods for type I/II and type III fractures are different. Type III fractures are often treated with a staged procedure, with external fixation as the primary surgery and ORIF or external fixation as the definitive treatment [15–17]. In contrast, type I/II fractures can be treated with debridement and ORIF in a one-stage manner [4, 18–20] with good results as suggested by McKee et al. [5] However, there were no studies comparing the difference of functional outcomes between OTA/AO type C, Gustilo type I/II, distal humerus open fractures and closed fractures treated by ORIF. Therefore, in this article, we reviewed Gustilo I and II, type C open fractures and closed fractures of the distal humerus treated in our hospital from 2014 to 2016 and compares the functional outcomes of the two groups treated by ORIF.

## Methods

### Patients

This was a retrospective, cohort, single-center study performed in our hospital. We obtained institutional review board approval for this retrospective investigation, and informed consent was obtained from each patient. The inclusion criteria were as follows: (1) patients with OTA/AO-C open and closed distal humerus fractures who were treated using ORIF from January 2014 to December 2016; (2) aged 18 years or older; and (3) follow-up period more than 2 year. The exclusion criteria were patients with (1) Gustilo type III open fractures; (2) pathological fractures; or (3) combined with other injuries.

**Fig. 1:**
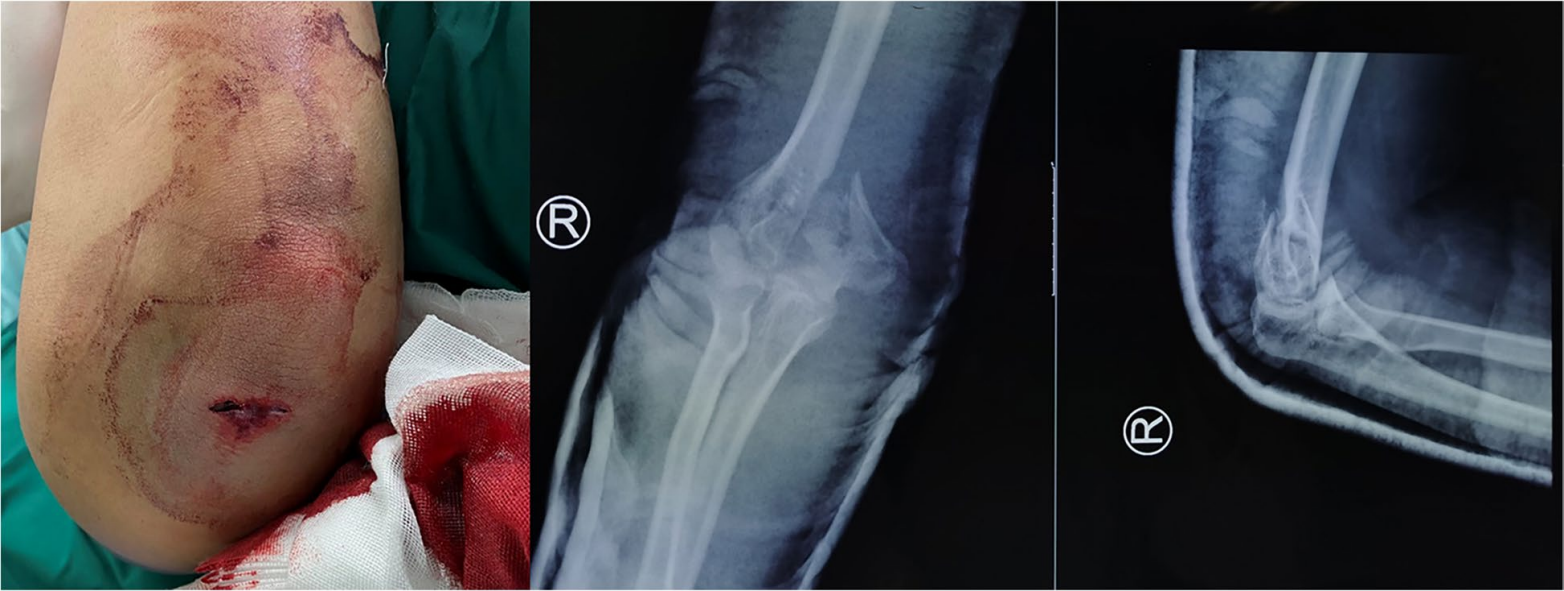
OTA/AO type C, Gustilo type I open fracture

All of the patients were examined in emergency to identify whether there were combined injuries and underwent radiographic imaging scans (anterior-posterior, lateral X-rays and CT scans) of the elbow to confirm the diagnosis and classification. Physical examinations were performed to determine whether there were neurovascular injuries. Patients with open fractures were treated with tetanus prophylaxis and antibiotics (cephalosporin or clindamycin, until the third day after ORIF) [21] and were immediately moved to the operating room for irrigation, debridement, and either immediate fixation or secondary fixation, depending on the condition of the patient’s soft tissue and doctor’s experiences (Fig. 1). For the patients who were treated by secondary fixation, the wounds were sutured, the elbows were immobilized using arm braces, and the wound dressings were changed daily for wound observing. If the wounds did not have swelling, erythema or drainage, the patients underwent ORIF 10–14 days later.

### Surgical technique

In ORIF surgery, the patients were anesthetized with brachial plexus block and operated through the posterior approach. After the skin was incised, the full-thickness flap was lifted, and the ulnar nerve was exposed and protected during the operation. If the joint needed to be fully exposed, olecranon osteotomy was performed. If the fracture could be directly reduced, the paratricipital approach involving both sides of the triceps was used. Reduction forceps and K-wires were used to maintain the reduction, then 2 anatomical locking plates and screws were used for fracture fixation. The plates were placed in parallel or vertically according to the surgeon’s preference [22]. After reduction and fixation were achieved, the ulnar nerve was transposed anteriorly to ensure that it was separated from the implant. The fascia and subcutaneous tissue were closed layer by layer, and the incision was closed after drainage. All of the patients were intravenously infused with second-generation cephalosporin antibiotics from 30 min before the internal fixation to 2 days after surgery.

### Postoperative treatment

**Fig. 2:**
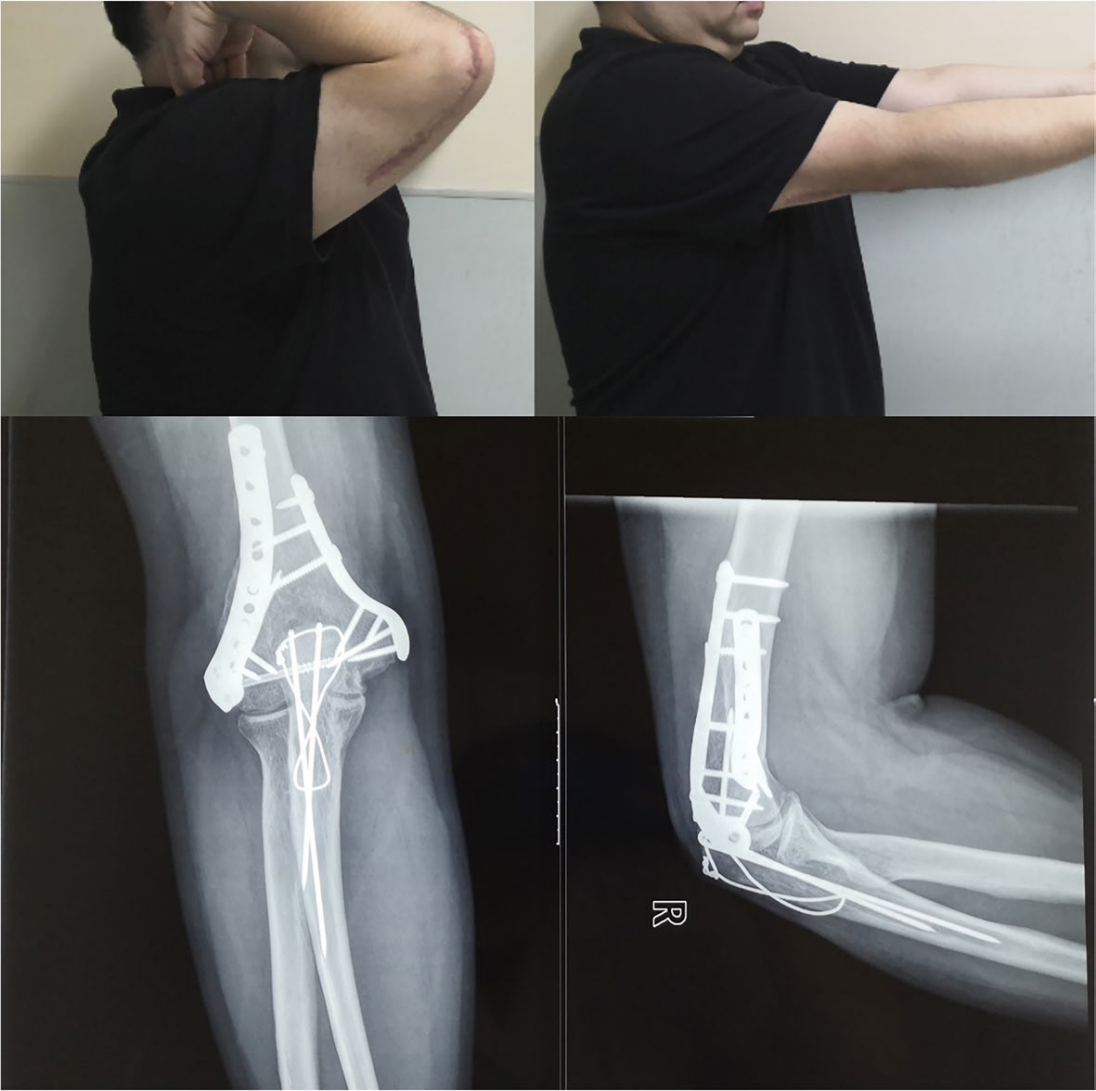
The elbow function and X-ray at the last follow-up

All of the patients started elbow rehabilitation on the second postoperative day. After the operation, the patients were followed up by a review clinic, and radiographs of the elbow were taken. The last follow-up was performed more than 2 year postoperatively (Fig. 2). In the last follow-up, we measured the ROM of the elbow, which is the primary outcome of the study. The secondary outcomes include: the Mayo Elbow Performance Score (MEPS), which was used to objectively evaluate the elbow with regard to 4 aspects: pain (45 points), range of motion (20 points), stability (10 points), and the ability to perform activities of daily living (25 points) [23]. The Disabilities of the Arm, Shoulder and Hand (DASH) questionnaire, which was used to subjectively evaluate elbow-related symptoms and disability [1, 24]. Complications such as infection, fracture nonunion, ulnar nerve symptoms, elbow stiffness, local irritation of the implant and secondary operations were recorded. Ulnar nerve injury was defined as a local sensory abnormality or weakened muscle strength after surgery [25]. Elbow stiffness was generally defined as an elbow range of motion (ROM) less than 100 degrees either in flexion-extension or in pronation-supination [26].

### Statistical analysis

Statistical analyses were performed using SPSS software for Windows (IBM SPSS Statistics, version 24; IBM, Armonk, NY, USA). For the quantitative variables, the descriptive statistics included means, medians, standard deviations, and ranges. The normally distributed data were compared using the t test for independent means. If the data were not normally distributed, the Wilcoxon rank-sum test was used. *P* < 0.05 was considered statistically significant.

## Result

The data for 64 patients with type C distal humerus fractures were collected from our database, and 25 cases were open fractures. The average follow-up duration was 35.1 ± 13.6 months(range, 25 to 46 months). The baseline characteristics [age, sex, body mass index (BMI), injury energy level, injury-to-operation time] were analyzed. There were no statistically significant differences between the open and closed fracture groups in the baseline characteristics (age, sex, injury energy level, injury-to-operation time, P>0.05). (Table 1).

**Table 1:** Comparison of the baseline characteristics between the two groups

Baseline characteristic		Open (*n* = 25)	Closed (*n* = 39)	*P* Value
Age[Mean ± SD]		43.2 ± 13.2	39.8 ± 15.2	0.351
Sex	Male	18	19	0.076
	Female	7	20	
Injury energy level	High energy	14	14	0.130
	Low energy	11	25	
Injury-to-operation time [M(P25, P75)](days)		6.0(1.0, 9.0)	5.0(3.0, 7.0)	0.648
Follow-up period		35.8 ± 11.6	34.7 ± 8.8	0.696

The perioperative and functional outcomes are reported in Table 2 and Table 3. The ROM of flexion and extension of open group is 118.9° ± 25.6°, while another group is 121.2° ± 25.5°(P>0.05). The ROM of rotation of the two groups are (150.4° ± 9.9°) and (153.5° ± 9.6°) (P>0.05). There were no statistically differences in the MEPS, DASH, hospitalization time, operation time, intraoperative blood loss, or treatment cost between the open group and the closed group (P>0.05). In terms of complications, there were no statistically significant differences in ulnar nerve injuries, elbow stiffness or local irritability in the region of internal fixation (*P* > 0.05). There was one case of nonunion of distal humerus fracture in closed group and one case of nonunion of olecranon osteotomy in open group. There was no patient had an infection.

**Table 2:** Comparison of the perioperative outcomes between the two groups

Functional outcome	Open	Closed	*P* Value
hospitalization time (days)	9.0(6.0, 13.0)	8.0(7.0, 10.0)	0.220
operation time (minutes)	165(120, 180)	150(120, 180)	0.306
intraoperative blood loss (ml)	100(100, 200)	100(100, 200)	0.852
treatment cost (K yuan)	102(87, 120)	93(85106)	0.645

**Table 3:** Comparison of the functional outcomes between the two groups

Functional outcome		Open	Closed	*P* Value
ROM of flexion and extension		118.9° ± 25.6°	121.2 ± 25.5	0.724
ROM of rotation		150.4° ± 9.9°	153.5° ± 9.6°	0.227
MEPS		89.2° ± 9.1°	89.5° ± 11.1°	0.914
DASH		4.2(0.8, 9.2)	2.5(0, 10.0)	0.530
Compli-cations	Ulnar nerve injury	6	9	0.932
	Elbow stiffness	3	5	0.923
	Local irritability of Internal fixation Nonunion	3	2	0.318
		1	1	0.747

## Discussion

The incidence of open distal humerus fractures is not high, but the fractures are difficult to treat, and the prognoses are very poor [27]. The fractures are difficult to treat because most of these injuries are caused by high-energy trauma, and the fractures are severely comminuted. In addition, compared with other joints, the elbow is a subcutaneous joint with thin soft tissue coverage and a complicated bone structure. If elbow injuries are not managed properly, patients will develop joint stiffness and other major complications. Even if good soft tissue treatment, anatomical reduction, and early rehabilitation have been achieved after surgery, postoperative upper limb dysfunction and related complications may still occur [28–30].

Chaudhary et al. [10] reported 8 cases of open intra-articular distal humerus fractures that were treated with open reduction and external fixation. The patients were followed up for an average of 11.4 months, they had an average ROM of 20°-120°, and 6 patients’ functional outcomes were excellent. Kömürcü et al. [31] reported 20 cases of open distal humerus fractures caused by gunshot wounds. The average follow-up duration was 34.3 months. There were 19 cases that were managed with external fixators. Regarding to MEPS, 8 cases had excellent final results, 7 cases had good results, and 4 cases had poor results. McKee et al. [5] reviewed 26 patients with open distal humerus fractures who were followed up for an average of 51 months. All the patients were treated with internal fixation after emergency debridement. The final average ROM was 97° (55° ~ 140°), and the MEPS was 79 (52 to 100). Complications included 1 case of deep infection and 2 cases of superficial infection. The patient with deep infection had Gustilo type III fractures. Kloen et al. [16] reported 16 cases of open intra-articular distal humeral fractures with temporary joint-spanning external fixation before internal fixation. The patients were followed up for an average of 35.2 months. All fractures united at an average of 5.2 months after internal fixation. No complications specifically related to the external fixation occurred. The DASH outcome score averaged 15.1, and 10 of 16 had an excellent/good outcome score. Min et al. [14] reported 14 patients with AO/OTA type C open distal humeral fractures and 14 closed fractures. For the open group, external fixation or ORIF were performed according to the injury after debridement treatment. For the closed group, ORIF was performed within 5 days after injury. The average follow-up time was 98.9 (52–160) weeks. The flexion-extension ROM was 108 ± 28.5° in the closed group and 82.5 ± 32.2° in the open group; the MEPS was 84.6 ± 19.3 in the closed group and 67.9 ± 22.4 in the open group. These differences between groups were statistically significant (*P* < 0.05).

These studies show that open distal humerus fractures can be treated with open reduction and internal or external fixation, and their prognoses are worse than those of closed fractures. However, previous studies have not considered the Gustilo subtypes. For Gustilo I, II and III fractures, the treatment methods and prognoses are significantly different. At present, the mainstream view is that Gustilo I and II fractures can be fixed in one surgery after debridement [4, 18–20, 32], and whether type III distal humerus fractures can be fixed in one surgery with debridement is still controversial [33–36]. Type III distal humerus fractures are mostly caused by high-energy trauma. The fractures are comminuted and accompanied by soft tissue defects, often requiring damage control surgery and multiple secondary soft tissue reconstruction treatments [37], with external fixation as the primary surgery and open reduction and internal fixation (ORIF) or external fixation as the definitive treatment [15, 16, 18]. Both treatments lead to a prolonged time of immobilization, which will result in functional loss of the involved elbow. In McKee’s study, severe complications such as deep infections occurred only in Gustilo III patients, and the author did not discuss type I/II patients separately [5]. Therefore, although this study showed that ORIF can be used to treat open distal humeral fractures, for patients with Gustilo type I and II fractures, whether ORIF can provide the same clinical effect a is not clear.

In summary, the clinical prognosis and risk of complications of Types I/II and III are significantly different, and previous clinical studies did not classify patients by Gustilo classes. Therefore, this study classified patients according to Gustilo classes and compared the treatment outcomes and prognoses of Gustilo I/II open fractures and closed fractures.

A total of 64 cases of type C distal humerus fractures were collected, and 25 cases were open fractures. There were no statistically differences in the ROM, MEPS, DASH score, hospitalization time, operation time, intraoperative blood loss, or treatment cost between the open group and the closed group. In terms of complications, there were no statistically significant differences in the rates for ulnar nerve injuries, elbow stiffness, nonunion or local irritation in the region of internal fixation.

Based on this result, for both AO/OTA type C Gustilo I/II open distal humerus fractures and closed distal humerus fractures, ORIF can be performed with the same approach after thorough debridement. Emergency ORIF can lead to early rehabilitation, so we recommend this procedure if possible in order to prevent elbow stiffness.

The advantage of this study is that it is the first to compare the efficacy of ORIF for AO/OTA Type C Gustilo I/II open distal humeral fractures and closed distal humeral fractures. However, this study also has some limitations: (1) this study is a retrospective rather than a prospective study, and the method of grouping can bias the results; (2) the Gustilo classification criteria is relatively broad and is determined by the surgeon’s judgment intraoperatively; (3) the sample size is larger than those in previous studies, but as open distal humeral fractures are still rare, the sample size is limited, which may have impact the results and statistical power; (4) there were differences between the open group and the closed group in the BMI, which may have impact the results. Therefore, additional large-scale studies are needed.

In summary, AO/OTA type C Gustilo I and II open distal humerus fractures treated with ORIF can exhibit satisfactory clinical results similar to those of closed distal humerus fractures in short terms. There were no significant differences in functional outcomes or complications between the open and closed groups. Moreover, the mean hospitalization time, operation time, intraoperative blood loss and treatment cost did not differ between groups. These results prove that the current treatment for this type of open fracture is reasonable.

## Data Availability

The datasets generated and/or analysed during the current study are not publicly available due to limitations of ethical approval involving the patient data and anonymity but are available from the corresponding author on reasonable request.

## Abbreviations

ORIF: Open reduction and internal fixation
MEPS: The mayo elbow performance score
DASH: The disabilities of the arm, shoulder and hand
ROM: Range of motion
BMI: Body mass index
